# 胸腔镜治疗≤10 mm非小细胞肺癌的临床研究

**DOI:** 10.3779/j.issn.1009-3419.2016.04.06

**Published:** 2016-04-20

**Authors:** 通 王, 少华 马, 天生 闫, 金涛 宋, 可毅 王, 未 贺, 洁 白

**Affiliations:** 100191 北京，北京大学第三医院胸外科 Department of Toracic Surgery, the Tird Hospital of Peking University, Beijing 100191, China

**Keywords:** 电视胸腔镜手术, 肺磨玻璃样结节, CT引导下Hook-wire定位, Video-assisted thoracic surgery, Ground glass opacity, CT-guided Hook-wire localization

## Abstract

**背景与目的:**

早期原发性非小细胞肺癌（non-small cell lung cancer, NSCLC）的手术切除及淋巴结切除的合理方式存在较大争议，本研究旨在探讨直径≤10 mm的原发NSCLC的微创切除及淋巴结切除的手术方式。

**方法:**

对2013年7月-2016年3月在我院接受电视胸腔镜手术（video-assisted thoracic surgery, VATS）治疗并有明确病理诊断为NSCLC的共46例患者的临床资料进行回顾性分析。所有患者术前行薄层计算机断层扫描（computed tomography, CT），实性结节5例，混合性磨玻璃结节（mixed ground-glass opacity, mGGO）23例，纯磨玻璃结节（pure ground-glass opacity, pGGO）18例。根据患者具体情况采用不同术式，包括VATS肺叶切除和系统性淋巴结清扫，VATS肺楔形切除和选择性淋巴结切除，VATS肺段切除和选择性淋巴结切除，或仅采用VATS肺楔形切除。其中7例术前行CT引导下Hook-wire定位。

**结果:**

VATS肺叶切除和系统性淋巴结清扫23例（mGGOs 15例，pGGOs 4例，实性结节4例），只有1例实性腺癌结节出现N2淋巴结转移，VATS肺楔形切除和选择性淋巴结切除5例（mGGOs 2例，pGGOs 3例）和VATS肺段切除和选择性淋巴结切除4例（mGGOs 2例，pGGOs 2例）均无淋巴结转移，仅采用VATS肺楔形切除14例（mGGOs 4例，pGGOs 9例，实性结节1例）。7例Hook-wire定位均成功。围手术期无重大并发症，随访1个月-26个月，平均（13.7±8.7）个月，无复发及转移。

**结论:**

直径≤10 mm以mGGO和pGGO为表现的原发性NSCLC淋巴结转移率低，术中可以不进行淋巴结的清扫，实性结节应选择性淋巴结切除或系统性淋巴结清扫。高龄和心肺功能差的患者可以选择楔形切除或肺段切除。术前运用Hook-wire定位安全有效，可为VATS提供便利。

## Abstract (en)

**Background and objective:**

The reasonable operational manner of non-small cell lung cancer (NSCLC) in early stage is in dispute. This clinical study is to investigate the operational manner of NSCLC 10 mm or less in diameter.

**Methods:**

The clinical datas of 46 cases with NSCLC 10 mm or less in diameter were retrospectively analyzed in our hospital from July 2013 to March 2016. Thin-section computed tomography (CT) was done on all cases with 46 pulmonary nodules (5 solid nodules, 23 mGGOs and 18 pGGOs). Lobectomy, wedge resection and segmentectomy with lymph node dissection may be performed in patients according to age or heart and lung function. CT-guided Hook-wire precise localization was done on 7 cases.

**Results:**

Lobectomy and systematic lymph node dissection under video-assisted thoracic surgery (VATS) were performed in patients with 23 pulmonary nodules (15 mGGOs, 4 pGGOs and 4 solid nodules), among wich, only one patient with N2 lymph node matastasis was found. Wedge resection and selective lymph node dissection under VATS were done in patients with 5 pulmonary nodules (2 mGGOs and 3 pGGOs), and segmentectomy and selective lymph node dissection under VATS were done in patients with 4 pulmonary nodules (2 mGGOs and 2 pGGOs), among wich, no patient with lymph node matastasis was found. CT-guided Hook-wire precise localization was done successfully on 7 cases.

**Conclusion:**

Usually NSCLC with pGGO and mGGO nodules 10 mm or less in diameter has no lymph node metastasis, therefore, systematic lymph node dissection may be not necessary. Selective lymph node dissection or systematic lymph node dissection should be performed in patients with solid nodules 10 mm or less in diameter. Wedge resection and segmentectomy may be performed in patients with advanced age or lower heart and lung function. The preoperative CT-guided Hook-wire localization for pulmonary nodules particularly for GGOs is an effective and safe technique to assist VATS resection of the GGOs.

随着影像技术的发展，以直径≤10 mm的肺部微小结节^[1]^及肺部磨玻璃结节（ground-glass opacity, GGO）为表现的肺腺癌的检出逐年增多^[2]^。根据2011年国际肺癌研究协会/美国胸科学会/欧洲呼吸学会对肺腺癌进行的新的病理分类^[3]^，直径≤10 mm的GGO可以为非典型腺瘤样增生（atypical adenomatoid hyperplasia, AAH）、原位腺癌（adenocarcioma *in situ*, AIS），亦可以是微浸润性腺癌（micro invasive adenocarcioma, MIA）甚至是浸润性腺癌（invasive adenocarcioma, IA）^[4]^，应当予以积极处理，但目前手术方式尚有争议。2013年7月-2016年3月我科对46例直径≤10 mm的原发性非小细胞肺癌（non-small cell lung cancer, NSCLC）（其中实性结节5例，GGOs 41例）在VATS下采用不同的手术方式，取得较好的临床效果，现将结果报道如下。

## 1 资料与方法

### 1.1 临床资料

2013年7月-2016年3月，共有46例单发、直径≤10 mm的原发性NSCLC患者在我院接受手术治疗。男性18例，女性28例，年龄22岁-73岁，平均（58.6±11.3）岁，右肺27例，左肺19例。直径5 mm-10 mm，平均（8.5±1.5）mm，实性结节5例，GGOs 41例，其中mGGOs 23例，pGGOs 18例。其中5例患者有肺外恶性肿瘤史，其余患者均体检发现，无恶性肿瘤病史。

### 1.2 术前定位

7例患者术前行CT引导下Hook-wire定位，然后施行VATS手术。

### 1.3 手术方法

采用静脉复合麻醉下双腔气管插管，健侧卧位。VATS手术时全面探查胸腔，以手指探查并结合影像或根据Hook-wire定位判断结节的具体位置，距离结节边缘2 cm再以直线型切割缝合器楔形切除病灶，送快速冰冻切片检查，根据病理结果及患者的心肺功能状态决定下一步手术方案。如为原发性NSCLC且患者能耐受手术，则继续行VATS肺叶切除术加系统淋巴结清扫或肺段切除加选择性淋巴结切除；若患者全身情况差，不能耐受肺叶切除术，则仅行肺楔形切除术和（或）淋巴结采样活检。

## 2 结果

### 2.1 定位效果

7例术前行CT引导下Hook-wire定位，均成功，无并发症。

### 2.2 手术统计

VATS肺叶切除和淋巴结清扫23例（mGGOs 15例，pGGOs 4例，实性结节4例），只有1例实性结节出现N2淋巴结转移，VATS肺楔形切除和选择性淋巴结切除5例（mGGOs 2例，pGGOs 3例）和VATS肺段切除和选择性淋巴结切除4例（mGGOs 2例，pGGOs 2例）均无淋巴结转移，仅采用VATS肺楔形切除14例（mGGOs 4例，pGGOs 9例，实性结节1例）。7例微小肺癌术前行计算机断层扫描（computed tomography, CT）引导下Hook-wire定位均成功。围手术期无重大并发症，恢复顺利，随访1个月-26个月，平均（13.7±8.7）个月，无复发及转移。

### 2.3 病理结果

23例mGGOs中，AIS 3例，IA 9例，MIA 2例，腺癌9例。18例pGGOs中，AIS 10例，MIA 4例，IA 2例，腺癌2例。5个实性结节中，肺原发性鳞状细胞癌3例，IA 2例。46例NSCLC中有淋巴结病理结果的32例中，Ia期T1N0M0 31例，IIIa期T1N2M0 1例（为实性结节，IA）。

## 3 讨论

标准肺叶切除+系统性淋巴结清扫术曾是早期原发NSCLC公认的标准手术治疗方式^[5, 6]^。随着Ⅰ期原发性NSCLC患者（特别是腺癌）的比例不断增加，传统经典的手术方式逐渐受到挑战。Ⅰ期特别是直径≤3 cm的NSCLC是否行系统性淋巴结清扫，还是采用淋巴结取样或选择性淋巴结清扫方式存有争议^[7, 8]^。近年来出现了早期NSCLC行部分肺叶切除和选择性淋巴结清扫术^[9, 10]^。直径小于2 cm的早期肺癌肺段切除和肺叶切除的远期生存率无差异^[11]^。由于操作快捷和相对较少的围手术期并发症及死亡率，VATS楔形切除常被用于亚肺叶切除治疗微小结节肺癌，主要用于高龄、合并心肺功能不全患者^[12-14]^。Miller等^[15]^专门对≤10 mm肿瘤施行肺叶切除、亚肺叶切除（肺段切除和楔形切除）进行对比，尽管肺叶切除组有明显的生存优势，但在亚肺叶切除的亚组分析中，无论是生存率优势还是局部复发率控制优势，都没有发现存在统计学差异。且系统性淋巴结清扫并未给早期肺癌患者带来更好的生存获益^[8, 16]^。Hashizume等^[17]^和Ohde等^[18]^研究结果显示，GGO所占比例≥50%的早期NSCLC中均未出现淋巴结转移，pTlNOM0期NSCLC患者的5年生存率达到95.7%，可以不行系统纵隔淋巴结清扫。章智荣等^[19]^研究显示，表现为pGGOs的34例Ⅰ期原发性NSCLC均未出现肺内（12组-14组）和肺外各组淋巴结转移。本组中经淋巴结采样和清扫的19例mGGOs和9例pGGOs，均未出现淋巴结转移。pGGOs的组织病理学类型常为AAH、AIS、MIA等，由于手术预后较好，病理类型为AIS、MIA的pGGOs的手术方式已逐渐推荐为亚肺叶切除（肺段或楔形切除）而非肺叶切除^[20]^。另有研究^[21, 22]^显示肺AIS多表现为pGGOs，生长缓慢，甚至可以在长达3年的时间里无明显变化，通常无淋巴结转移，手术后效果良好，若完整切除可以达到治愈，5年生存率达100%。对于肺AIS通常采用VATS肺楔形切除或肺段切除，其有效率和肺叶切除相当，完全可以替代传统肺叶切除^[23]^。但对于混合型病变，其恶性程度与GGO和结节实性成分所占比例有关。实性成分比例在提示病变的性质和治疗预后有着非常重要的意义，实性成分的增多一定程度上提示着恶性病变存在进展可能^[24, 25]^。本研究对直径≤10 mm肺微小结节的32例原发性NSCLC进行淋巴结清扫或采样后，只有1例实性结节出现淋巴结转移。该患者病变位于右肺上叶，肿瘤大小为9 mm，术后病理类型为IA，共清扫淋巴结16枚（肺门淋巴结6枚，纵隔淋巴结10枚），只有1枚纵隔淋巴结转移。章智荣等^[19]^研究显示，47例mGGOs中仅有1例出现淋巴结转移，病变位于右肺下叶前基底段，实性成分占70%，GGO成分占30%，肿瘤大小为1.1 cm×0.8 cm，术后病理为中分化腺癌，共清扫淋巴结7枚，其中肺门淋巴结转移1枚。因此，mGGOs实性成分增多应考虑行选择性淋巴结清扫或系统性淋巴结清扫^[19]^。NSCLC淋巴结转移率随着肿瘤直径的增大而增加^[26, 27]^，手术应根据患者情况进行手术方式的选择并附加淋巴结清扫或采样^[28]^。直径≤10 mm以GGOs为表现的原发性NSCLC淋巴结转移率低，术中可以不进行淋巴结的清扫，实性结节应选择性淋巴结切除或系统性淋巴结清扫。

肺楔形切除与肺叶切除相比有较高的局部复发率，主要与肺叶内肿瘤残留、肺叶内淋巴结转移、切缘阳性等有关^[29-31]^。相较于VATS楔形切除，VATS解剖性肺段切除具有的生存优势和较少的局部复发率主要和以下几个技术特点密切相关：①严格按解剖面进行切除；②切除范围足够大，距肿瘤边缘2 cm以上；③肺实质内引流淋巴组织的切除。VATS解剖性肺段切除较VATS楔形切除更接近肿瘤手术切除要求，更好地解决了肿瘤局部复发的难题。无论选择何种亚肺叶切除，建议术中评估淋巴结状态，以取得更全面的肿瘤分期，以便和肺叶切除疗效有更完整对比^[32, 33]^。本组采用楔形切除和肺段切除的23个病例，随访1个月-26个月，尚未出现局部复发病例，有待进一步随访观察。我们的经验是楔形切除应距肿物边缘2 cm以上，以保证切缘阴性。另外对于较小的GGOs，术前可以采用CT引导下Hook-wire定位，以方便确定GGOs位置。这种方法成功率高，并发症少^[34, 35]^。
